# Infections in Healthcare Workers in Germany—22-Year Time Trends †

**DOI:** 10.3390/ijerph15122656

**Published:** 2018-11-26

**Authors:** Albert Nienhaus

**Affiliations:** 1 Competence Centre for Epidemiology and Health Services Research for Healthcare Professionals (CVcare), University Medical Centre Hamburg-Eppendorf (UKE), Martinistraße 52, 20246 Hamburg, Germany; albert.nienhaus@bgw-online.de; Tel.: +49-40-7410-24727; 2 Department of Occupational Medicine, Hazardous Substances and Public Health, Institution for Statutory Accident Insurance and Prevention in the Health and Welfare Services (BGW), Hamburg, Germany

**Keywords:** health worker, infection, hepatitis, tuberculosis, occupational disease

## Abstract

Health workers (HWs) run an increased risk of infection. The standardised data set of an accident insurer was used to analyse the time trends of infection-related claims and confirmed occupational diseases (ODs) in HWs. The numbers of claims and confirmed claims for different infections were analysed for the years 1996 to 2017. The rate of claims and confirmed ODs were calculated per 100,000 full-time workers. The number of claims was relatively stable over time. However, the rate per 100,000 full-time workers decreased from 25.2 to 15.4. The decrease was most pronounced for hepatitis B and hepatitis C infections, which were the most frequent infections for which claims were made at the start of the period. In 2017, tuberculosis (TB)-related claims were more frequent than those related to blood-borne virus infections. However, the growing number of TB claims does not reflect an increased infection risk, but rather improved methods for the diagnosis of latent TB infection (LTBI). Measures to prevent blood-borne virus infections in HWs were successful in the last 22 years, but attention should be paid to newly emerging infections.

## 1. Introduction

Health workers (HWs) run an increased risk of contracting infectious diseases. This increased risk is well documented for a variety of old infections such as tuberculosis [1,2,3], blood-borne virus infections such as hepatitis C [4], and influenza [5]. However, it also exists for new infections such as Severe Acute Respiratory Syndrom (SARS) [6,7,8,9], the H1N1-pandemic in 2009 [10,11], and multidrug resistant bacteria like Methicillin-Resistant *Staphylococcus Aureus* (MRSA) [12]. For the USA researchers estimated the excess death rate due to infections in HWs as 9–29 per one million HWs [13]. Therefore, efforts are made to protect HWs against infections [14]. The Needlestick Safety and Prevention Act (NSPA) enacted in the USA in 2000 was a landmark in protecting HWs from blood-borne virus infections. The NSPA mandates that employers select safer needle devices and train employees on the proper use of all engineering and work practice controls [15]. The American example was followed by European Union regulations encouraging the use of safe instruments in healthcare that were soon implemented in Germany [16,17]. Health workers are consulted on infection prevention measures and examined for infectious diseases on a regular basis. If a vaccination for a particular infection such as hepatitis B exists and if HWs are at risk of this infection, vaccination is offered at the employer’s expense and the occupational health physician is responsible for recommending and performing the vaccination [18]. Depending on the risk assessment, the employer has to provide personal protective equipment (PPE). Consideration of different approaches for the protection of HWs gives rise to the question of how well these regulations and obligations protect HWs against infections at the workplace. One possible way to answer this question is by analysing the time trends of occupational diseases (ODs) caused by infections in HWs [19]. In Germany, as in many other countries, infection in a HW can be recognised as an OD and be compensated accordingly. For example, from 1996 to 2013 a total of 1121 hepatitis C infections in HWs were recognised as ODs in Germany and a total of EUR 52 million was paid to compensate for the loss of ability to work. A further EUR 36 million was paid for medical treatment of these HWs between 2000 and 2014 [20]. 

Assuming that regulations and safety and health recommendations help to improve the protection of HWs against infections, the number of infectious diseases in HWs should have decreased. In order to test this hypothesis, the number of claims relating to infectious diseases in HWs and the number of confirmed cases were analysed over a period of 22 years in Germany.

## 2. Materials and Methods

A descriptive retrospective observational study was carried out about the mandatory claims and the confirmed ODs of infectious diseases of HWs, covering the last 22 years using insurance data.

The Institution for Statutory Accident Insurance and Prevention in Healthcare and Welfare (BGW—*Berufsgenossenschaft für Gesundheitswesen und Wohlfahrtspflege*) is the compensation board for all private healthcare and welfare providers in Germany. A total of 630,000 companies with about eight million insured persons are covered by the BGW. In addition to HWs, the insured persons can be hairdressers, social workers, and volunteers. In Germany, about one-third of all hospitals are public and therefore covered by other state-held insurances. However, in recent years some public hospitals were privatised and changed to having the BGW as their insurer. The BGW’s standardised database of compensation claims relating to infectious disease was used for this analysis. Data on infections caused by animals or tropical diseases was not included. The database makes it possible to distinguish between the most frequent infectious diseases. The date of registration of a claim and the decision whether the claim is confirmed as an OD are documented in this standardised data set. As the decision on the claim may not be reached in the year when the claim is filed, or because several decisions can be taken due to the appeal of a decision, the number of claims decided differs from the number of claims filed in a particular year. Therefore, a valid confirmation rate cannot be calculated per year. An average confirmation rate was calculated by summarising all registered claims and all confirmed cases for the total period. This does not provide an exact confirmation rate but an estimate that should be rather close to the true value.

It is compulsory for physicians to report suspected cases of ODs after first diagnosis. In the standardised data set, a further distinction is made between ODs that are mandatorily reportable and those that are not. It is not mandatory to report contacts with infectious patients or materials. Therefore, they were excluded from this analysis. Time trends from 1996 to 2017 were analysed for mandatory claims only. A distinction can be made between tuberculosis (TB), latent tuberculosis infection (LTBI), methicillin-resistant *Staphylococcus aureus* (MRSA), epidemic keratoconjunctivitis, influenza, human immunodeficiency virus/acquired immunodeficiency syndrome (HIV/AIDS), hepatitis B virus (HBV), and hepatitis C virus (HCV). All other infections were combined in the category ‘Other Infections’. Latent tuberculosis infection is considered present if the interferon-γ release assay (IGRA) is positive, and active TB is excluded by X-ray. Infections with parasites are not considered, as in the case of these infections the data set does not allow for a clear distinction between contact with patients with parasitic infection and actual infection.

The rate of claims per 100,000 full-time workers as well as the rate of confirmed ODs per 100,000 full-time workers was calculated. Two part-time workers working 50% of the normal working time of 39 h per week were regarded as one full-time worker. Volunteers, such as first aids or social assistance workers, are not paid for their work. As they do not work regular hours, they are not included in the calculation of full-time workers. Therefore, the number of full-time workers is far lower than the number of insured persons.

## 3. Results

In the last 22 years, the number of infections notified was rather stable, fluctuating between 646 cases in 2010 and 1066 cases in 2004 (Table 1). On average, 810 cases were filed each year. However, when the growing number of HWs insured by the BGW is taken into account, the notification rate decreased. The number of full-time workers insured by the BGW increased from 3,238,000 in 1996 to 4,935,000 in 2017. This is an increase of 52%. In 1996, 25.2 claims per 100,000 full-time workers were filed. In 2017, this rate decreased by 39% to 15.4 (Table 1). The rate of confirmed ODs per 100,000 did not change much between 1996 and 2017 (7.0 versus 6.9 per 100,000). The average confirmation rate was 33.0%.

**Figure 1:**
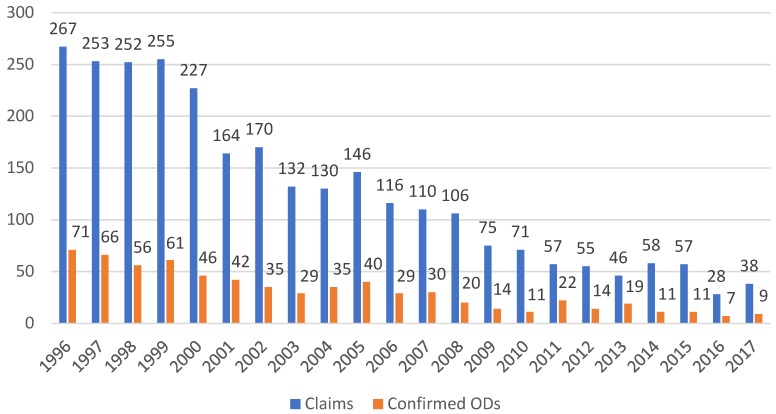
Hepatitis B: Claims and confirmed ODs from 1996 to 2017. Average confirmation rate for hepatitis B virus (HBV): 24.3% (total number of claims: 2817; total number of confirmed ODs: 685).

**Figure 2:**
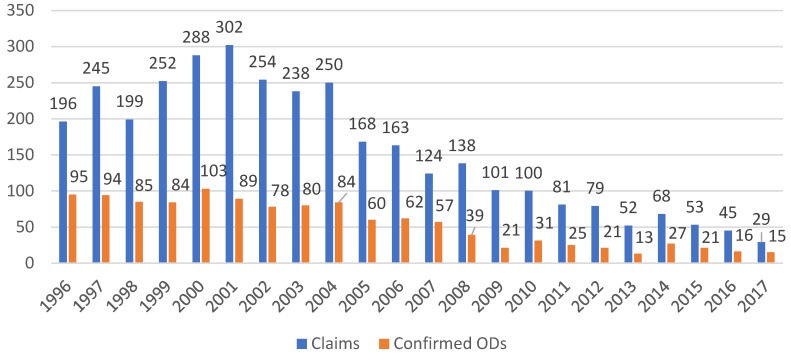
Hepatitis C: Claims and confirmed ODs from 1996 to 2017. Average confirmation rate for hepatitis C virus (HCV): 39.7% (total number of claims: 4761; total number of confirmed ODs: 1361).

The decrease in notified and confirmed cases was most pronounced for blood-borne virus infections. Notified hepatitis B decreased by about 90%, from 267 cases in 1996 to 38 cases in 2017 (Figure 1). The same trend was observed for confirmed claims, with numbers decreasing from 71 to 9. The rate per 100,000 full-time workers decreased from 1996 to 2017 from 8.2 to 0.8 for claims and from 2.2 to 0.2 for hepatitis B-related ODs (Table 2). For hepatitis C, the pattern was different. In the first six years the number of notifications increased from 196 in 1996 to 302 cases in 2001. Thereafter, the numbers decreased steadily to 29 in 2017 (Figure 2). The number of confirmed cases was roughly stable between 1996 and 2000 but then started to decrease, from 89 cases in 2001 to 15 cases in 2017. The rate per 100,000 full-time workers decreased from 1996 to 2017 from 6.1 to 0.6 for claims and from 2.9 to 0.3 for hepatitis C-related ODs (Table 2). The average confirmation rate was 24.3% for hepatitis B and 39.7% for hepatitis C.

For HIV, the number of notified and confirmed cases was low throughout the whole period (between 2 and 12 claims per year). In total, 141 claims were registered and 34 cases confirmed as OD. A peak of 10 confirmed cases was recorded in 2011. Otherwise, no time trend is apparent (Table 3). The average confirmation rate for HIV was 24.1%.

A total of 124 influenza-related claims were registered throughout the whole period and 48 were confirmed as OD (Table 3). There was a spike during the H1N1 pandemic in 2009 (53 claims). The average number of claims per year increased from 1.1 before 2009 to 6.6 after 2009. The average confirmation rate for influenza was 38.7%.

Epidemic keratoconjunctivitis was the cause of 4 to 50 claims per year, with no obvious time trend. The confirmation rate was 55.9% (Table 3). Claims for cytomegaly were rather rare (one to nine cases per year) and the average confirmation rate was 21.0%.

Unlike blood-borne virus infections, the time trend for TB showed an increase. Though there was considerable variation, the number of claims more than doubled between 1996 and 2017 (173 versus 473 claims) when LTBI and active TB cases are taken into account together. Confirmed claims showed an even steeper increase, from 43 in 1996 to 299 cases in 2017. When distinguishing between active TB and LTBI, an increase was mainly observed for LTBI (Table 4). For active TB, the number of claims increased from 173 in 1996 to 222 in 2017 (an increase of 28%), although the rate of claims per 100,000 full-time workers decreased from 5.3 to 4.5. However, the rate per 100,000 full-time workers for active TB as OD increased from 1.3 to 2.0, indicating that the confirmation rate increased over time. Notification of LTBI only started in 2006 (20 claims) and increased to 201 claims in 2017. The rate per 100,000 full-time workers for LTBI as OD increased from 0.1 in 2006 to 4.1 in 2017. The average confirmation rate for active TB was 37% and for LTBI was 64.0%.

The number of MRSA cases notified varied. In 2006, the year when MRSA was first recorded separately, 114 cases were registered and one case was confirmed as OD (Table 5). In 2017, 39 cases were registered and one case was confirmed. In general, the number of notified cases was lower in the last five years than in previous years. Regardless of the number of notified cases, only a few cases were confirmed (9.2%) and there was no evident time trend.

The number of all other infections was 157 in 1996 or 19.2% of all claims. In 2017, this number increased to 203 or 26.7% of all infection related claims. The average confirmation rate was 12.3%, the lowest of all infections (data not shown).

## 4. Discussion

All types of ODs considered, the incidence of infections in German HWs seems to have declined in the last 22 years. The time trend was quite different for blood-borne virus infections than for TB. While an increase was observed for TB, the decrease in HBV and HCV infections explains the overall decrease in ODs over time. Advances in HW vaccinations against HBV infection are the most likely explanation for the decrease of HBV infection in HWs [21]. As vaccination is not mandatory, different steps were taken to improve the vaccination rate in HWs. There is provision for a mandatory consultation with occupational physicians at least every third year for HWs at risk [18]. After a needle stick injury (NSI), vaccination status is checked and vaccination is started if needed. In addition, a vaccination campaign was mounted by BGW in 2002. As well as sponsoring vaccination and thereby covering some of the cost for the employer, BGW carried out an information campaign focusing on the responsibility of employers and employees for preventing nosocomial transmission. This campaign was not evaluated, but it is reasonable to assume that it helped to increase awareness and therefore improve the vaccination rate among HWs. Two recent surveys found HBV vaccination rates of 70% in nurses [22] and 94% in dentists [23]. Therefore, a high vaccination rate in HWs in Germany can be assumed, although no systematic analysis exists. Germany is a low incidence country for HBV infections. In 2001, a total of 3876 new HBV infections were registered for the general population. This number decreased to 1684 in 2012. Thereafter the number of registered HBV infections increased again to 3622 cases [24]. The decline of HBV infection in HWs can therefore only partly be explained by the epidemiologic situation in the general population.

Vaccination cannot explain the decrease in HCV infection in HWs, as no vaccination exists as of yet. The decrease of HCV infection in HWs is partly due to the decline of HCV infection among the general population. Germany is a low incidence country for HCV infections. In 2004, a total of 9,044 new HCV infections were registered for the general population. This number decreased to 4,798 (53.1%) in 2017 [25]. Therefore, the decrease in the general population was less pronounced than the one for HWs. Most likely this is due to the use of safe instruments, double gloves, safe disposal of used instruments, and general awareness of HWs [26]. In Germany, the number of NSI claims filed with the BGW increased [27]. We assume that this is not due to a real increase in NSIs but to the increased awareness that NSIs might have dangerous consequences. This interpretation is supported by the decrease in HCV infections recorded as suspected OD. In addition, 30% to 50% of NSI claims filed nowadays are caused by needles for subcutaneous injections [28]. These devices pose a lower infection risk than blood collecting devices [29].

In 22 years, 141 HIV/AIDS-related claims were registered and 34 claims confirmed, which gives a confirmation rate of only 24.1%. After a NSI, the risk for HIV infection is considered to be lower than for HBV or HCV infections [14]. There are additional requirements for confirming an OD, such as proving that the index patient is HIV positive, or treatment of risk groups combined with a severe NSI. Successful post-exposure prophylaxis (PEP) might be a reason for the low number of claims. However, we do not know how often PEP is performed in Germany. A survey found that PEP for HIV was offered in 8% of all NSI cases and no infection was observed subsequently [30].

Despite a sharp decline in TB incidence in the general population over the last six decades, TB as an occupational disease increased in Germany [31]. At first glance, this observation is contradictory. However, this development is caused by two factors unrelated to the actual infection risk or the recent increase in TB among the general population due to the migration crisis in 2015 [32]. First, the perception of infection risks for HWs changed. Until 2003, only a few healthcare tasks were believed to be associated with an increased risk of infection for HWs. In 2003, an expert panel reconsidered these assumptions and came to the conclusion that all HWs working at entry points to the healthcare system where patients are not yet diagnosed (e.g., emergency rooms, surgery, nursing homes) are at increased risk of TB infection. Furthermore, delayed diagnosis of TB in patients is a particular risk factor for TB transmission in hospitals and other health facilities [33]. After the likelihood of accepting TB as an OD increased, the number of claims increased. Second, with the introduction of IGRA the diagnosis of LTBI became more reliable [34,35,36,37,38]. Therefore, from 2006 the number of claims relating to LTBI started to increase. Whenever preventive treatment is performed in these cases, the costs can be covered by the insurance. As such, the increased number of cases does not necessarily reflect an increased risk, but increased protection of HWs. 

The number of influenza cases filed is surprisingly low. This is most likely due to the fact that the accident insurance covers long-term effects on workers’ health. However, this should not discourage any attempts to improve coverage of HW vaccinations [39]. It is well proven that influenza vaccinations in HWs reduces working time lost [40]. To which extent vaccination of HWs will protect patients from nosocomial transmission of influenza from HWs is debated [41,42]. 

A particular problem are infections that are troublesome during pregnancy. There are few cases of cytomegalovirus (CMV) infection filed as OD. On the other hand, there are strict regulations concerning infectious diseases in pregnancy. For example, pregnant women with no natural immunisation against CMV need to stop working with young children in kindergartens or hospitals [43,44]. It is beyond the scope of this analysis to decide whether these regulations are effective, as no systematic evaluation exists. 

Epidemic keratoconjunctivitis is caused by adenovirus. It typically starts with a unilateral foreign body sensation. Within a few hours or days, it develops into bilateral keratoconjunctivitis with marked chemosis, epiphora, and photophobia. Visual impairment can persist for months because of corneal infiltrates and irregular astigmatism. As nosocomial spread of adenoviruses is relatively common, ophthalmologists and their assistants are at increased risk of infection [45,46]. This is mirrored by the high claim confirmation rate (55.9%). Transmission risk can be reduced by hygienic measures, including conscientious hand and surface disinfection [45].

Methicillin-resistant Staphylococcus aureus is seldom filed as an OD. Most HWs colonised with MRSA will not develop a MRSA-related infection. Following German Occupational Disease regulations, colonisation is not considered as a disease and can therefore not be recognised as OD. This explains why the confirmation rate of MRSA-related claims is low. Recent surveys found colonisation rates of 1% to 3.2% in HWs in nursery care and patient transport workers. The average rate is 1.5% [47,48,49]. In a review comprising eight German surveys, an average colonisation rate of 3.1% for HWs was described. This rate was well above the colonisation rate in the general population (0.2%). Nurses were more often affected than physicians [12]. As colonisation of a HW with MRSA is not covered by accident insurance, occupational health physicians are reluctant to screen HWs for MRSA due to not knowing how to manage cases of MRSA-positive HWs who are not only transitorily colonised [50,51,52]. In order to prevent transmission of MRSA from HWs to patients, regulations are needed for the screening of HWs and the management of MRSA-positive HWs.

For this analysis, routine data are used. These data do not distinguish HWs with and without migration background. HWs with migration background have a higher risk for infection (i.e., the prevalence of LTBI in HWs with migration background is twice as high as in HWs born in Germany) [38]. However, the effect of migration on the time trend of infections as OD cannot be studied with the data available. Another problem might result from underreporting of infections as ODs. Underreporting is evident for influenza. For active TB, underreporting is the explanation for fewer cases reported before 2003, as physicians knew that the chances for the confirmation of TB as OD were low. Even though the degree of underreporting cannot be estimated, underreporting is an unlikely explanation for the positive trend in HBV and HCV infections in HWs. A further shortcoming of this analysis is the lack of information on workplace and job titles. However, from earlier projects it can be concluded that about 70% of all ODs in HWs concern hospitals or nursing homes [53]. About 75% of all HWs with OD-related infections are women [20].

## 5. Conclusions

The number of ODs caused by infections in HWs decreased over the last 22 years in Germany. This decrease was most pronounced for blood-borne virus infections. The increase in TB-related claims is due to a changed risk perception and improved methods for diagnosing LTBI. Nevertheless, attention needs to be given to preventing infections in HWs in order to prevent nosocomial transmission. 

## Figures and Tables

**Table 1:** Claims and confirmed occupational diseases (ODs) from 1996 to 2017.

Year	Full-Time Workers in Thousands (*N*)	All Claims (*N*)	Claims per 100,000 Full-Time Workers	ODs Confirmed	ODs per 100,000 Full-Time Workers
1996	3238	816	25.2	226	7.0
1997	3157	884	28.0	238	7.5
1998	2888	849	29.4	246	8.5
1999	2831	889	31.4	252	8.9
2000	2990	882	29.5	216	7.2
2001	3019	791	26.2	186	6.2
2002	3189	810	25.4	232	7.3
2003	3627	809	22.3	200	5.5
2004	4213	1,066	25.3	199	4.7
2005	3431	988	28.8	296	8.6
2006	3595	798	22.2	239	6.7
2007	3589	786	21.9	211	5.9
2008	3773	717	19.0	200	5.3
2009	4010	818	20.4	255	6.4
2010	4089	646	15.8	304	7.4
2011	4299	761	17.7	324	7.5
2012	4406	727	16.5	368	8.4
2013	4531	879	19.4	422	9.3
2014	4585	729	15.9	360	7.9
2015	4587	711	15.5	301	6.6
2016	4753	789	16.6	290	6.1
2017	4935	760	15.4	340	6.9
Total		17,905		5905	

The average number of claims per year is 810. The average confirmation rate of all claims is 33.0%.

**Table 2:** Hepatitis B and C claims and confirmed occupational diseases (ODs) per 100.000 full-time workers from 1996 to 2017.

Year	Hepatitis B		Hepatitis C	
	Claims per 100,000 full-time workers	ODs per 100,000 full-time workers	Claims per 100,000 full-time workers	ODs per 100,000 full-time workers
1996	8.3	2.2	6.1	2.9
1997	8.0	2.1	7.8	3.0
1998	8.7	1.9	6.9	2.9
1999	9.0	2.2	8.9	3.0
2000	7.6	1.5	9.6	3.4
2001	5.4	1.4	10.0	3.0
2002	5.3	1.1	8.0	2.5
2003	3.6	0.8	6.6	2.2
2004	3.1	0.8	5.9	2.0
2005	4.3	1.2	4.9	1.8
2006	3.2	0.8	4.5	1.7
2007	3.1	0.8	3.5	1.6
2008	2.8	0.5	3.7	1.0
2009	1.9	0.4	2.5	0.5
2010	1.7	0.3	2.5	0.8
2011	1.3	0.5	1.9	0.6
2012	1.3	0.3	1.8	0.5
2013	1.0	0.4	1.2	0.3
2014	1.3	0.2	1.5	0.6
2015	1.2	0.2	1.2	0.5
2016	0.6	0.2	1.0	0.3
2017	0.8	0.2	0.6	0.3

**Table 3:** Claims and confirmed occupational diseases (ODs) for human immunodeficiency virus/acquired immunodeficiency syndrome (HIV/AIDS), influenza, keratoconjunctivitis epidemica, and cytomegaly.

	HIV/AIDS		Epidemic Kerato-Conjunctivitis		Cytomegaly		Influenza	
Year	Claims (*N*)	Confirmed ODs (*N*)	Claims (*N*)	Confirmed ODs (*N*)	Claims (*N*)	Confirmed ODs (*N*)	Claims (*N*)	Confirmed ODs (*N*)
1996	8	4	8	20	5	0	2	0
1997	3	1	13	8	4	1	1	0
1998	5	5	44	20	4	1	0	1
1999	6	3	50	27	9	2	2	0
2000	12	1	44	23	2	0	1	1
2001	8	0	9	16	7	0	0	0
2002	9	1	19	14	5	1	6	0
2003	3	0	18	4	2	3	1	0
2004	11	2	7	5	2	0	0	0
2005	9	2	16	6	1	0	0	0
2006	12	0	23	12	3	0	0	0
2007	4	4	24	8	4	0	0	0
2008	11	2	10	5	2	1	1	0
2009	5	0	23	4	5	2	53	32
2010	5	1	16	18	4	1	5	4
2011	10	10	21	5	4	0	12	3
2012	2	3	42	21	1	0	2	0
2013	3	6	35	22	3	1	11	1
2014	5	0	15	7	1	0	9	1
2015	3	0	13	4	5	1	6	0
2016	3	5	5	3	3	2	8	4
2017	4	2	4	1	5	1	4	1
Total	141	34	456	255	81	17	124	48

Average confirmation rate: HIV/AIDS 24.1%, influenza 38.7%, keratoconjunctivitis 55.9%, cytomegaly 21.0%.

**Table 4:** Claims and confirmed occupational diseases (ODs) of tuberculosis (TB) and latent TB infection (LTBI) from 1996 to 2017.

	TB				LTBI				All			
Year	Claims (*N*)	A	Confirmed ODs (*N*)	B	Claims (*N*)	A	Confirmed ODs (*N*)	B	Claims (*N*)	A	Confirmed ODs (*N*)	B
1996	173	5.3	43	1.3	-				173	5.3	43	1.3
1997	201	6.4	51	1.6	-				201	6.4	51	1.6
1998	155	5.4	48	1.7	-				155	5.4	48	1.7
1999	124	4.4	47	1.7	-				124	4.4	47	1.7
2000	136	4.6	24	0.8	-				136	4.6	24	0.8
2001	125	4.1	29	1.0	-				125	4.1	29	1.0
2002	125	3.9	33	1.0	-				125	3.9	33	1.0
2003	134	3.7	40	1.1	-				134	3.7	40	1.1
2004	191	4.5	50	1.2	-				191	4.5	50	1.2
2005	234	6.8	99	2.9	-				234	6.8	99	2.9
2006	175	4.9	83	2.3	20	0.6	5	0.1	195	5.4	88	2.5
2007	253	7.1	56	1.6	33	0.9	6	0.2	286	8.0	62	1.7
2008	195	5.2	73	1.9	40	1.1	17	0.5	235	6.2	90	2.4
2009	124	3.1	61	1.5	187	4.7	65	1.6	311	7.8	126	3.1
2010	102	2.5	72	1.8	199	4.9	125	3.1	301	7.4	197	4.8
2011	115	2.7	68	1.6	285	6.6	166	3.9	400	9.3	234	5.4
2012	146	3.3	66	1.5	227	5.2	179	4.1	373	8.5	245	5.6
2013	160	3.5	77	1.7	383	8.5	244	5.4	543	12.0	321	7.1
2014	94	2.1	81	1.8	297	6.5	205	4.5	391	8.5	286	6.2
2015	155	3.4	57	1.2	235	5.1	178	3.9	390	8.5	235	5.1
2016	235	4.9	80	1.7	268	5.6	161	3.4	503	10.6	241	5.1
2017	222	4.5	98	2.0	251	5.1	201	4.1	473	9.6	299	6.1
Total	3574		1336		2425		1,553		5999		3889	

A: Claims per 100,000 full-time workers; B: ODs per 100,000 full-time workers. Average confirmation rate of TB: 37.4%; average confirmation rate of LTBI: 64.0%.

**Table 5:** Claims and confirmed occupational diseases (ODs) relating to methicillin-resistant *Staphylococcus aureus* (MRSA) from 2006 to 2017.

	MRSA	
Year	Claims (*N*)	Confirmed ODs (*N*)
2006	114	1
2007	88	5
2008	98	11
2009	102	8
2010	49	9
2011	52	9
2012	60	9
2013	44	7
2014	58	5
2015	54	7
2016	47	2
2017	39	1
Total	805	74

Average confirmation rate of MRSA: 9.2%.
